# An avenue to miscarriage: a case report

**DOI:** 10.1007/s12024-022-00484-3

**Published:** 2022-06-07

**Authors:** S. Grey, J. Lartey, J. Millington, R. Vardon, J. Crane, J. Preuß-Wössner, C. Buschmann

**Affiliations:** 1 Libertas Chambers, London, UK; 2 Lartey & Co Solicitors, London, UK; 3 Millington Hingley Ltd, London, UK; 4 18 St John Street Chambers, Manchester, UK; 5 The Queens University, Belfast, Northern Ireland; 6 Institute of Legal Medicine, University Hospital Schleswig-Holstein, Kiel/Lübeck, Germany

**Keywords:** Crime scene investigation, Bloodstain pattern analysis, Ability to act, Forensic strategy, Miscarriage of justice

## Abstract

Two men were wrongfully convicted of murder in 2017 and sentenced to life imprisonment. After a physical altercation inside a flat, the victim (A) was found dead approximately 60 m away outside a residential address. He had sustained a number of injuries including a stab wound to the left side of his neck which was found to have divided the right carotid artery. The location where A was found was not regarded as a crime scene and not subjected to a specialist forensic examination by scientists as it was assumed that the fatal injury was sustained in the flat. The pathologist, who subsequently carried out the autopsy on A, was not asked to attend the scene. A review of the blood distribution at the scene in conjunction with the pathology findings indicated however that the fatal neck wound had been inflicted outside the flat, near to where the victim was found. An appeal against the convictions for murder was upheld in 2021 and a re-trial ordered. Following this second trial, both accused were acquitted of murder and released from custody. The new pathology and blood pattern evidence introduced at the second trial was a major part of the defense strategy which led to the acquittal of the accused. The case illustrates that a more inclusive and detailed crime scene strategy had been undertaken, including an assessment of the bloodstains present, in conjunction with discussion with the pathologist, then the likelihood is that the two men subsequently charged with murder would have been eliminated as suspects and a miscarriage of justice would have been avoided.

## Case details

The victim A lived in a multi-occupancy flat in London with other Polish nationals, including one of the defendants B. A co-defendant, C, lived in an adjacent street and a further defendant, D, had been staying in the victim’s flat at the time of the incident which occurred in the early hours of the morning in August 2016. A was assaulted in the front room of his flat which was used as his bedroom. This room was on the ground floor of the property, above a basement flat, and was accessed via a set of steps leading up to the communal entrance of the property. A was attacked with a knife in the front room of his flat and sustained bleeding injuries. His cries for help were heard by other occupants of the house who went to his flat and knocked on the door which was opened by D. Following this initial assault, A escaped from the flat through the front bay window. On exiting the window, he then had to traverse the narrow window ledge and climb down onto the front steps leading onto the pavement. Defendants B and C were observed on CCTV leaving the flat and walking away in the opposite direction to A. The other occupant of the flat, D, followed A. Short time later, B returned to the property and into A’s room where he moved around and stood on a sofa which was positioned under the bay window. In doing so, he stepped in A’s blood, which was present on the floor, and transferred footwear marks. In the meantime, A had collapsed outside the front door of a residential property approximately 60 m away in another avenue. His blood-soaked T-shirt was found beside his body. He had been heard shouting for help in the street and is believed to have crouched behind a parked car. The occupant of the residential property, who was wakened by a disturbance outside, found A collapsed in a pool of blood on his doorstep.

## Crime scene examination strategy

The scene within the front room of the deceased’s flat was identified after the police followed a trail of blood spots from where the body of A was found. It was evident that the trail of blood back to the flat had a significant impact on how the crime scene examination strategy developed. The strategy employed by the police was based on the assumption that (i) A had been assaulted exclusively in the front room of the flat; (ii) all of his injuries had been sustained there; and (iii) the trail of blood demonstrated the route that he took after he left the property through the bay window, pausing behind the parked car before making his way into the avenue where he eventually collapsed outside a residential property. Part of his journey was corroborated by private CCTV footage. This showed that his hands were down by his side—an observation that had a significant impact on the expected blood loss from the fatal neck injury. These erroneous initial assumptions made by the police were then conveyed to others, including forensic science experts who attended the scene and documented bloodstaining, etc. within the flat and on the window and communal steps. Although the attending scientists were aware that a blood trail led from the flat to the area where the body was found, this area was not subject to specialist scientific examination and only general scene photographs were taken.

## Crime scene (inside the flat–Fig. 1a**–**b)

**Fig. 1:**
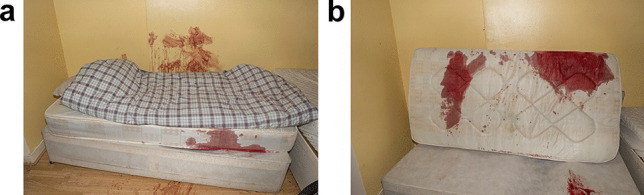
Bloodstained wall (**a**) and blood-soaked mattress (**b**) inside the flat

The inside of the flat was examined by a bloodstain scientist. The distribution of blood comprised a combination of transfer and spattered bloodstains. There was clear evidence of some form of assault having taken place on or near the bed with heavy saturated bloodstaining on the mattress. There was transfer bloodstaining, mixed with hair, on the wall behind the bed indicating that the deceased had sustained some injuries to his head—this was confirmed at autopsy by the finding of an extensive incised wound across the top and back of the scalp. The extent and severity of the scalp injuries were sufficient to account for the heavy bloodstaining within the flat.

## Crime scene (outside the flat–Fig. 2a)

**Fig. 2:**
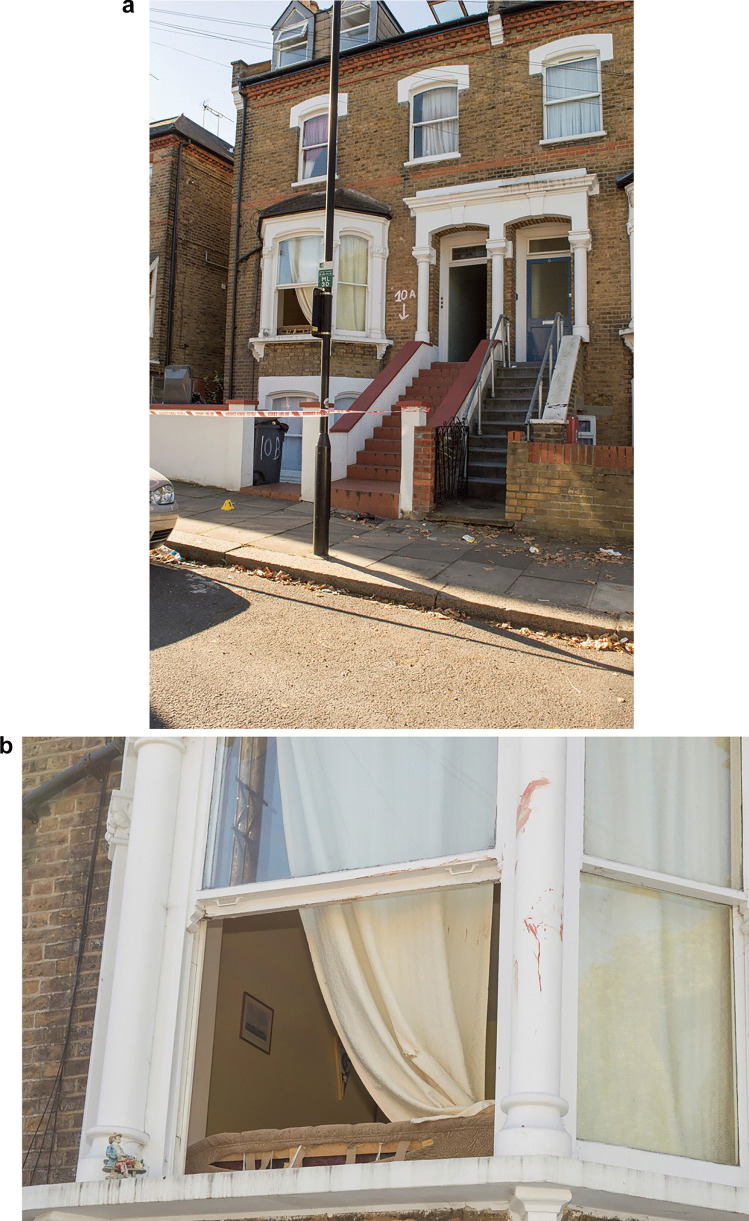
Outside the flat (**a**), a few bloodstains around the bay window of the flat where the later victim escaped (**b**)

The area immediately outside the flat was also examined by a bloodstain scientist. A limited number of drip stains were identified in the area of the bay window and transfer bloodstaining was present on the underside of the window frame. The findings corroborated the view that **A**, having already been injured, had left the property from the bay window. However, there was no apparent expirated bloodstaining or evidence of heavy bleeding from a severed major blood vessel.

## Crime scene (route taken by A–Fig. 2b)

All areas beyond the immediate vicinity of the flat were not subjected to specialist forensic examination. Scene photographs showed a small number of drip stains on the pavement along the initial part of the route taken by A after he left the flat. The relative paucity of dripped blood suggested that A was not bleeding heavily at this time. Bloodstains on the road at the rear of the parked car and on the rear bumper of the vehicle corroborated the view that A had stopped there and that he was bleeding more freely at that time.

## Crime scene (avenue where deceased was found–Fig. 3a–c)

**Fig. 3:**
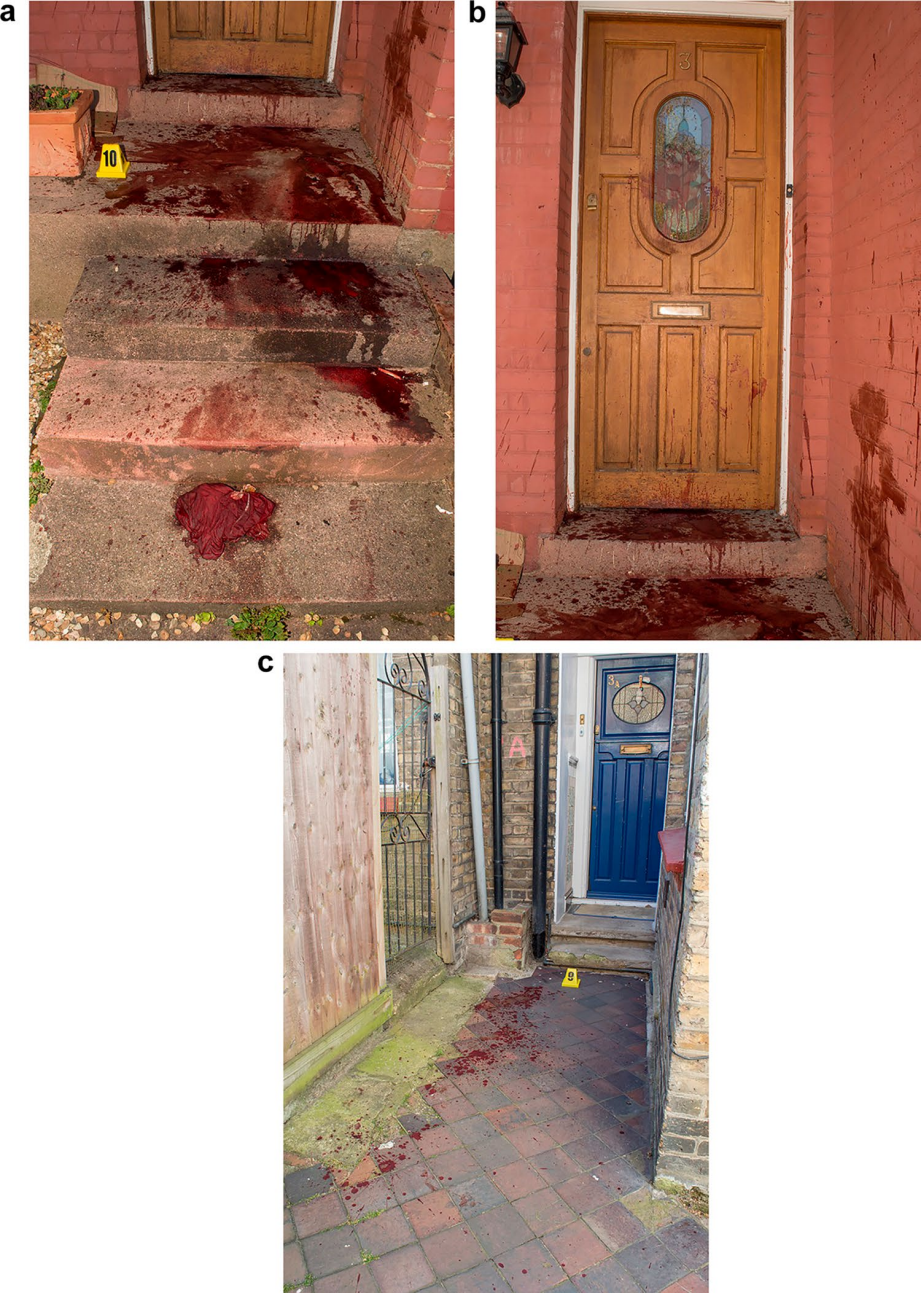
Location of the infliction of the fatal wound with marked increase in dripped bloodstains (**a**) and heavy bloodstaining where the victim was found dead a few metres away (**b**, **c**)

The avenue, and an alleyway leading from it, was not subjected to a specialist forensic examination. Photographs demonstrated a substantial increase in bloodstaining in an alley off this avenue and large volume drips of blood could be seen on the ground and walls. The blood would appear to have dripped directly from **A**’s injuries and/or from surfaces that had become saturated with blood. In places, the blood drops were present in clusters and were associated with pronounced secondary spatter, a feature that is typically associated with prolonged or large volume blood loss and can indicate ‘pause points’ and/or locations where a volume of blood has been suddenly released. In summary, the blood findings in the alley suggested that **A** had been there for a period of time and that blood loss had markedly increased there. Extremely heavy bloodstaining, including extensive pooling and drip patterns, was present outside the residential property where **A** was found slumped against a wall. Heavy bloodstaining was present on the door of the property including some expirated blood that showed pronounced bubbling. A number of large directional drops of blood were seen on the walls on each side of the door and appeared to comprise vomited/coughed blood (from **A**’s mouth or neck injury), along with probable projected blood lost from his neck wound or cast from surfaces wet with blood. The features collectively indicated that **A** had sustained the fatal stab injury to his neck at this location resulting in significant amounts of blood entering his airways.

## Autopsy findings (Fig. 4)

**Fig. 4:**
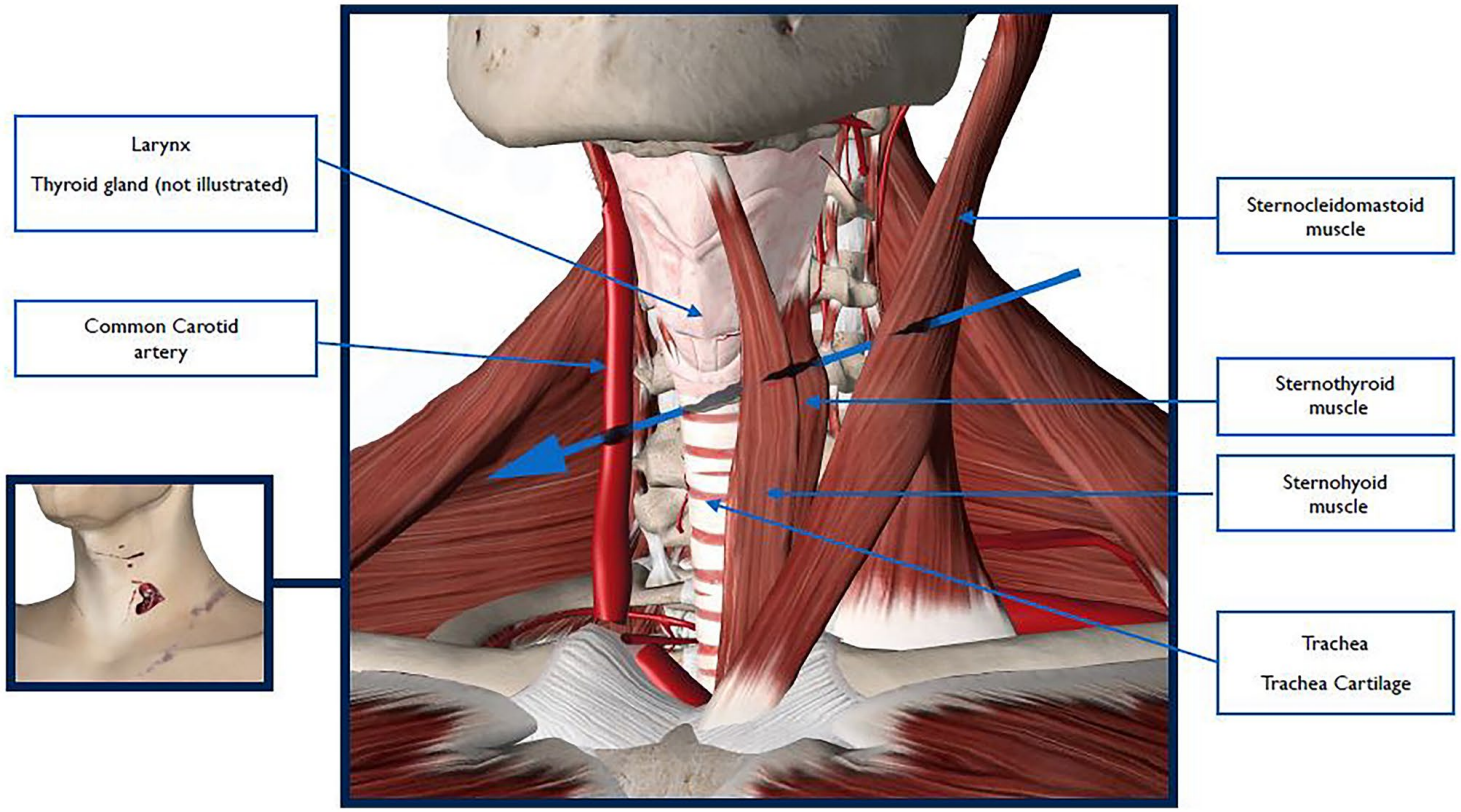
Schematic drawing of the fatal wound

The deceased was a healthy 25-year-old male (height 184 cm and weighing 72 kg). He had sustained a number of incised wounds to his head, neck, left shoulder, left wrist and right forearm. Sharp force trauma to the back of the head corresponded to bloodstains of Fig. 1a and b in the flat. The fatal wound was a 14-cm deep stab wound to the left side of his neck which passed across the neck through the muscles and trachea to the right side where it had divided the right carotid artery. Further details of this arterial stab wound were not provided in the autopsy report. This was deemed the fatal injury and would have caused rapid but not immediate death. The deceased had also sustained a number of blunt force injuries consistent with him having been assaulted. Blood had been aspirated into his air passages and lungs. Toxicology revealed some cannabis and amphetamine but the levels detected were not deemed significant.

## First criminal trial

Following the police investigation, persons B, C and D were charged with murder and sent for trial. Defendant B told the jury that he had seen D with a knife attacking the deceased in the front room and hence had left but had returned a short time later to look for the victim providing an explanation for his partial footwear marks in A’s blood. The prosecution however sought to attribute this as evidence that he had been present in the room at the time of the attack. Unfortunately, defendants B and C lied to the police and this tainted their later testimony and undermined their credibility as witnesses in respect of the actions of D. None of the medical or scientific experts involved in the initial investigation appeared to have questioned the assumption that an individual with a fatal stab wound to the neck would have been capable of climbing out through a window, negotiating a narrow ledge, jumping onto steps and then running over 60 m before collapsing and without leaving a significant trail of blood en route. It was ostensibly accepted that a young, healthy male could carry out strenuous physical activity after the fatal injury was sustained and that the lack of blood along the escape route could be accounted for by him using his T-shirt to stem the bleeding. After a trial lasting 6 weeks and based on the assumption that the fatal stab wound and all the other injuries were sustained whilst A was inside the flat when all 3 defendants were present, the jury convicted B and C of murder by a majority 10−2 verdict. The jury were unable to reach a verdict in respect of D who they had been told was of good character. He was subsequently re-tried but once again the jury failed to reach a verdict. The crown offered no further evidence against him and a ‘not guilty’ verdict was entered.

## Post-conviction review of the scientific findings

Following the verdict, work of newly instructed experts challenged the basis on which the crime scene examination strategy had been progressed. A review of the pathology findings, blood distribution and CCTV footage casts serious doubt on the assumption that the fatal neck wound had been sustained inside the flat. The opinion of the newly instructed pathologists was that A’s fatal neck injury would have bled massively immediately after infliction and would have rapidly limited his ability to carry out purposeful movements and activities. The right carotid artery had been divided and this would have resulted in rapid and massive blood loss leading to hypovolaemic shock and collapse, probably within a few minutes. Even if, as suggested, A was able to stem the flow of blood by applying pressure to his neck, blood would have entered the partially divided trachea causing difficulty breathing. This, and an incision through the larynx, would have rendered him incapable of screaming loudly despite a witness hearing loud screams after he had left the flat. Also, it would have been expected that expirated blood patterns would have been found inside the flat and in the area around the exit route through the window if the airways were filled with blood—these areas were subjected to a full examination by bloodstain pattern scientists and no expirated blood was found. It was the opinion of the new pathology experts that all the bloodstaining inside the flat could be accounted for by the scalp and back wounds which would have bled considerably. The forensic biologist who reviewed the case was of the opinion that the relative amount of bloodstaining outside the flat, as observed from the scene photographs, was initially limited but then increased substantially in the alley close to where A was found. Pronounced drip patterns were present there, and although in themselves could not be used to identify the actions or scenario which led to their development, they indicated that A was bleeding extensively at this location. Heavy areas of bloodstaining on and around the door of the property where A had collapsed and died, including pronounced exhaled blood from his mouth or neck (from the laryngeal and tracheal wounds), indicated that A had sustained the major stab wound to his neck and that significant amounts of blood were entering his airways at this time. Given A’s activities over the proposed timescale and the absence of significant bloodstaining, commensurate with the neck wound, around the bay window and behind the parked car, in combination with the paucity of dripped blood en route to the alley, all cast doubt on the initial assumption of where the fatal attack took place. The conclusion that the fatal stab wound was inflicted in the alley some distance from the flat effectively excluded defendants B and C as being responsible.

## Appellate proceedings

In January 2021, the convictions of B and C were quashed and a re-trial ordered.

## 2nd trial

B and C stood trial at the Central Criminal Court in August 2021. The new scientific and pathology evidence was presented to the court and, as a result, both men were acquitted of murder. They were released from custody and were free to return home to their families in Poland.

## Discussion

The incapacity to act differs individually and is dependent on age, pre-existing illnesses and injury pattern. A differentiation is made between immediate, faster, delayed, restricted and lack of incapacity to act, depending on the injury pattern [1, 2]. Immediate incapacity to act is given if the brain as the central control unit of the entire organism is affected. Rapid inability to act results from damage to vital structures such as the heart, pulmonary trunk or aorta, when severe blood loss causes rapid circulatory depression with subsequent hypoxia of the brain, whilst delayed inability to act is caused by damage to large vessels or organs (liver, lungs, kidneys). In forensic literature, however, exceptions are repeatedly reported when victims were able, for example, to shoot themselves in the head several times [3–5], to run long distances [6, 7] or to respond to fire despite gunshot-related heart rupture [8].

In this case, the new pathology and scientific evidence was critical in reconstructing the sequence of events leading up to the death of A. The initial reconstruction of events as determined by the police investigation team, including the pathologist and scientific experts initially instructed was, as was later agreed, badly flawed. The underlying forensic strategy was based on the assumption that all of A’s wounds, including the fatal stab wound to the neck, had been inflicted within the flat. This error had a significant impact on how the investigation developed. Because the crime scene and forensic personnel did not question this assumption, on which their instructions were based, a detailed examination of the bloodstaining on the roadway, or in the alley, did not take place. That A would have been capable of climbing out of the window, negotiating the narrow window ledge and then jumping onto the steps before running a significant distance before collapsing was accepted without question and consequently it was felt that there was no reason to examine the bloodstains along that route in any detail. The absence of discernible bloodstains along the initial section of road leading from the flat and the relative paucity of blood around the bay window were explained by A being able to staunch the flow of blood from his injuries. The flawed strategy was inappropriately reinforced and inadequately challenged leading to a miscarriage of justice. There was no doubt that the attack on A started inside his flat as demonstrated by bloodstaining on the floor, walls and bed but it was also assumed, and not challenged, that the fatal neck wound was also inflicted here. This assumption allowed the crown to attribute responsibility for the killing to all 3 men who had been present in the flat. The review conducted by the newly instructed pathology and scientific experts challenged the basis on which the initial crime scene examination had progressed. The outcomes of their review were then subject to a joint conference between the pathologists and forensic scientists instructed on behalf of the prosecution and defense with the production of agreed joint statements. In this case, 6 independent medical and scientific experts, from 3 jurisdictions, concluded that A’s fatal injury had been sustained outside the flat and in the avenue or alley, 60 m away. This joint opinion was a crucial piece of evidence at the re-trial and, in no small measure, contributed to the acquittal of both B and C both of whom had already spent 4 years in prison. The development of the forensic strategy in this case started with an assumption that was not challenged or reviewed during the course of the investigation and nurtured an environment for confirmation bias. A more inclusive crime scene examination strategy would have mitigated the damage that those assumptions had on the evolving investigation. The importance of specialist scene examination by forensic experts in those cases where blood pattern analysis is required cannot be over emphasised [9]. It is also essential that forensic scientists and pathologists work together sharing information to assess the significance of wounds and their relevance to scene interpretation. In this case, the focus of the investigation was misplaced and the scientific findings so fragmented that a miscarriage of justice was inevitable.

## Key points

1. Crime scene examination prior to autopsy should, where possible, be carried in suspected cases of homicide.
2. Scene examination and interpretation is crucial for forensic pathologists. Reliance solely on isolated autopsy findings may result in loss of key evidence.
3. In suspected homicide cases, it is important that pathologists take into consideration scene findings and work in conjunction with forensic scientists when drawing up reports and conclusions.
4. Whilst bloodstain pattern analysis is carried out by crime scene investigators and forensic scientists, it is recommended that forensic pathologists have specialised training in this field.
5. Ability to act remarkably differs in individual subjects with regard to age, pre-existing illnesses and other confounding factors. Nonetheless, general statements can be made with respect to individual injury patterns and circumstances of the respective case.
